# Diagnostic challenges in dengue encephalitis: subtle imaging findings and initially negative laboratory test in a kidney transplant recipient

**DOI:** 10.1055/s-0045-1812888

**Published:** 2026-02-04

**Authors:** Juan Sebastián Sánchez León, Vanessa Dantas de Andrade, Amanda Fernandes Klajn Maycá, Carolina Matte Dagostini, Marcela Nataly Parra Alvarez, Marlise Ribeiro de Castro

**Affiliations:** 1 Universidade Federal de Ciências da Saúde de Porto Alegre, Porto Alegre RS, Brazil.; 2 Santa Casa de Misericória de Porto Alegre, Porto Alegre RS, Brazil.

**Keywords:** Dengue, Encephalitis, Transplants, Immunosuppression Therapy

## Abstract

Dengue encephalitis is an uncommon neurological complication of dengue virus infection. In immunocompromised patients, such as solid organ transplant recipients, the clinical presentation may be atypical and nonspecific, posing significant diagnostic and therapeutic challenges. We report a clinical case and use it as a framework to discuss key considerations for suspicion of dengue encephalitis. Topics include optimal timing for clinical suspicion, essential differential diagnoses, and the most appropriate diagnostic strategies. Emphasis is placed on the correct interpretation of cerebrospinal fluid findings and recognition of subtle brain magnetic resonance imaging changes that may support the diagnosis. The discussion also reviews current evidence on dengue encephalitis in immunocompromised populations, highlighting implications for timely diagnosis and management in this vulnerable group.

**TEACHING POINTS**

1. For dengue diagnosis, the choice of test should be guided by the day of symptom onset. Nonstructural protein 1 (NS1) sensitivity progressively declines, particularly after day 5.
2. On magnetic resonance imaging (MRI) scans, abnormalities may be heterogeneous and sometimes subtle—for example, loss of cerebrospinal fluid (CSF) suppression—reflecting altered CSF composition in inflammatory or infectious conditions.
3. Neurological management requires close monitoring and tailored symptomatic treatment throughout the disease course, with the goal of preventing complications and optimizing outcomes.

## CLINICAL VIGNETTE

A 56-year-old man with a history of 2 kidney transplantations (2006 and 2013) having chronic immunosuppressive therapy with mycophenolate and tacrolimus. He presented to the hospital with a 5-day history of fever, asthenia, and myalgias. He was confused, somnolent, showing generalized muscle rigidity and myoclonus on admission, without other changes in the physical examination.

An infectious etiology was first considered because of febrile syndrome. Meanwhile, baseline laboratory tests, including complete blood count and metabolic panel, were performed and showed no important abnormalities. A dengue NS1 antigen test was performed on day 5 of symptoms, due to regional epidemiological context, and the result was negative.

Considering the patient's immunosuppressed status and the persistence of neurological symptoms, a lumbar puncture was indicated to investigate a potential central nervous system (CNS) infection. The CSF analysis revealed elevated protein levels and mild lymphocytic pleocytosis ( Table 1 ), suggesting a central inflammatory process. The bacterial culture and Gram stain were both negative.

**Table 1:** Lumbar puncture cerebrospinal fluid results

Cerebrospinal fluid	Lactate	Glucose	Proteins	Erythrocytes	Leucocytes	Lymphocytes
**Sample 1**	2,2 mmol/l	62 mg/dl	175 mg/dl	1	5	87%
**Sample 2**	2,2 mmol/l	73 mg/dl	175 mg/dl	30	40	86%

**Figure 1:**
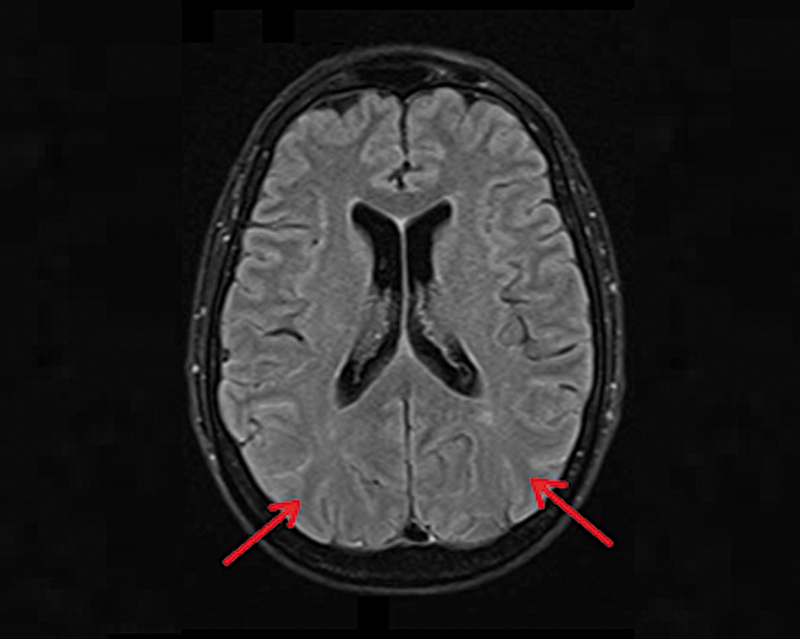
Magnetic resonance imaging brain T2-FLAIR sequence. Areas with a mild loss of signal saturation of cerebrospinal fluid in certain cortical sulci of both cerebral hemispheres.

Brain MRI showed findings consistent with viral encephalitis, including mild loss of CSF signal suppression in the bilateral cortical sulci ( Figure 1 ), supporting the hypothesis of CNS involvement likely due to an infectious or parainfectious process. An electroencephalogram (EEG) was also performed, demonstrating disorganized cerebral activity, absence of normal physiological rhythms, and diffuse 4-to-5 Hz theta activity throughout the recording.

A subsequent CSF analysis revealed increased leukocytes compared with the previous examination, with other abnormalities remaining similar ( Table 1 ). The polymerase chain reaction (PCR) for herpesviruses on this sample was negative. Given the ongoing suspicion of a viral etiology and the known reduced sensitivity of the NS1 antigen test beyond day 5 of symptoms, dengue immunoglobulin M (IgM) serology was ordered on day 7 of symptom onset—2 days after the initial NS1 testing. The result was positive and was subsequently confirmed with a repeat serology 2 days later.

After 7 days of hospitalization—11 days after symptom onset, and with management focused on supportive treatment, the patient showed spontaneous clinical improvement, becoming alert, coherent, and experiencing complete resolution of muscle rigidity and myoclonus. On hospital day 13, he was discharged with full recovery of neurological function ( Table 2 ).

**Table 2:** Clinical course and dengue test results

Symptoms and test results	First symptoms		Hospitalization and neurological onset. Negative Dengue NS1		Dengue IgG: 0.3 (< 1.1) IgM: 1.7 (< 1.1)		Dengue IgM 4.0 (< 1.1)		Improvement of symptoms		Hospital discharge day 13
**DAY**	1	2–4	5	6	7	8	9	10	11	12–16	17

Abbreviation: NS1, nonstructural protein 1.

## FROM PRESENTATION TO RESOLUTION: LESSONS LEARNED

### How frequent is dengue-associated encephalitis, when should it be suspected, and what are the underlying pathophysiological mechanisms?

Dengue, an arboviral infection mainly transmitted by the *Aedes aegypti* mosquito, continues to be a major public health problem in tropical and subtropical areas. Globally, more than 390 million infections and around 96 million symptomatic cases occur annually. 1 Most cases are indeed self-limiting, but severe manifestations—especially those associated with neurological complications—pose a considerable clinical burden. Neurological involvement reported in dengue ranges from 0.5 to 5.4% in Southeast Asia; some Brazilian studies revealed incidence up to 5%. 1 2 3 The most important symptoms include altered mental status, seizures, focal neurological deficits, and signs of meningeal or radicular irritation. 4 5 6 7

There are several mechanisms proposed for the neurological complications of dengue, either due to hepatic dysfunction, metabolic derangements, or direct neurotrophic actions of the virus. Neuromuscular involvement can present as either Guillain-Barré syndrome or transient myositis; neuro-ophthalmic manifestations may impair vision. 2 7 8 In addition to direct viral neuroinvasion, delayed immune-mediated complications may occur 1- to 3-weeks after infection. Proposed mechanisms include molecular mimicry and antibody cross-reactivity with myelin components. Examples of such complications include acute disseminated encephalomyelitis (ADEM), myelitis, Guillain-Barré syndrome, neuromyelitis optica, and postinfectious encephalopathy. 7

Dengue encephalitis—inflammation of the brain resulting in neurological dysfunction—represents a significant proportion, ranging from 4 to 47%, of encephalitis-like illness admissions in endemic areas. 1 It is one of the most serious manifestations of dengue and has a heterogeneous range of symptoms, usually characterized by headache, seizures, altered consciousness, confusion, behavioral changes, focal deficits, and stiff neck. 6 7 8

### Can the presentation of dengue encephalitis differ in immunocompromised patients, such as renal transplant recipients?

Solid organ transplant recipients and other immunocompromised individuals are particularly vulnerable to atypical and severe forms of dengue, including CNS involvement. In this population, the clinical course may be more aggressive, given impaired viral clearance and altered inflammatory responses. Classical symptoms, such as fever and myalgia, may be blunted or absent in this population. The limited data on dengue encephalitis in transplant recipients underscores the need for enhanced surveillance and customized preventive strategies in this high-risk population. 8 9

Disordered immune regulation results in impaired T cell and natural killer (NK) cell activities, leading to ineffective viral clearance and increased levels of neuroinflammation. As a result, neurological manifestations including confusion, rigidity, and myoclonus may be more pronounced, frequently making diagnosis challenging and delaying therapeutic measures. 8

### What are the primary differential diagnoses that should be excluded?

In our clinical case, given the acute neurological presentation, several differential diagnoses were systematically considered. Herpes simplex virus (HSV) encephalitis was initially considered as the main diagnostic hypothesis, given the presence of fever, altered mental status, and abnormal motor signs. However, HSV PCR was negative in the CSF, there were no suggestive findings on the cranial resonance, and Gram stains and cultures were also negative, which excluded classical viral encephalitis. Based on lack of systemic signs of infection, negative laboratory results, and lack of septic foci, bacterial encephalitis and atypical meningitis were also excluded.

Metabolic or toxic encephalopathy (often encountered in immunosuppressed persons) was deemed improbable, given that an extensive laboratory work demonstrated no disturbances of electrolytes, altered liver function or presence of toxic agents. In view of clinical progression with rigidity, myoclonus and focal seizures, an immune-mediated postinfectious process such ADEM was considered. However, the latter is predominantly seen in pediatric populations, and the lack of white matter lesions on MRI, combined with spontaneous clinical improvement in the absence of immunomodulatory therapy, rendered this diagnosis less plausible. Considering the clinical course, the exclusion of other etiologies, and the epidemiological factors, the most likely diagnosis was neurological symptoms associated with dengue.

### How should the diagnosis be established, and which investigations should be requested?

Laboratory confirmation of dengue infection primarily relies on serological testing, 10 particularly IgM and immunoglobulin G (IgG) antibodies that signify recent and past infections, respectively. The NS1 antigen detection test is especially valuable in the early phase, preceding antibody production, as it demonstrates the highest sensitivity within the first 3 days after symptom onset, with a significant decline in sensitivity beyond day 5. Typically, IgM becomes detectable within 5 to 7 days of symptom onset, whereas IgG appears later and may indicate secondary infection. 11 12 In cases presenting with neurological manifestations, comprehensive evaluation including neuroimaging and CSF analysis is essential. 5

The use of MRI scans plays a key role in identifying affected regions, which most often include the basal ganglia, thalami, temporal lobes, hippocampi, cerebellum, and subcortical white matter. Additional reported imaging findings include meningeal enhancement, symmetrical edema, and cerebral microhemorrhages. 5 11 12 Also, more subtle abnormalities may be present, as in this case, and should not be overlooked. These include areas of loss of CSF signal saturation in the cortical sulci, which can be observed in a variety of conditions, including inflammatory disorders like encephalitis. 13

Importantly, the most common finding in CSF analysis is pleocytosis, which is generally lymphocytic. However, the results are often nonspecific or even normal, and a negative finding does not exclude the diagnosis. 5 13 14

Definitive diagnosis is based on the detection of the virus in CSF, for which PCR is the gold standard. Although IgM detection can have high specificity, its sensitivity may be low. As such, the diagnosis should be made by combining serum and CSF tests, which must be requested according to the day of evolution of the disease. 11 12

In transplantation receivers, the strategies for diagnosis should account for the changes to the immune response, which could mask classic clinical features and prolong the seroconversion time. A multimodal diagnostic strategy, combining NS1 antigen testing, IgM/IgG serology, and reverse transcription polymerase chain reaction (RT-PCR), leads to improved diagnostic accuracy is therefore recommended. 4 In the case presented, the limited availability of PCR-based diagnostic techniques for dengue at our institution was the primary reason for their nonutilization.

Several criteria can be used to define a case of dengue encephalitis, as shown in Table 3 . 11 14 15

**Table 3:** Criteria for case definition of a patient with dengue encephalitis 11 14

Evidence of dengue infection	Positive NS1 antigen, RT-PCR, or dengue-specific IgM in serum or CSF*
Compatible clinical presentation	Acute febrile illness** + neurological symptoms: altered mental status, seizures, and/or focal neurological signs not attributable to other causes
Exclusion of other causes of viral encephalitis and encephalopathy	

Abbreviation: CSF, cerebrospinal fluid; IgM, immunoglobulin M; NS1, nonstructural protein 1; RT-PCR, reverse transcription polymerase chain reaction.

Notes: *CSF pleocytosis (usually lymphocytic) and compatible alterations on MRI can strengthen clinical suspicion. **It may be absent in immunosuppressed patients.

### What is the prognosis in immunocompromised patients, and what is the recommended treatment approach?

The prognostic outcomes in this population are generally poorer, and current evidence remains inconclusive regarding the use of immunomodulatory therapies—specifically corticosteroids and intravenous immunoglobulin (IVIG). 14 Classical clinical features are rare and there aren't targeted treatment options, which underscores the importance of individualized supportive care and a high index of diagnostic suspicion. 5 7 In this case, immunomodulatory therapy was not administered due to a lack of robust supporting evidence or established clinical guidelines. Therefore, management was primarily supportive, emphasizing close neurological monitoring and symptomatic treatment. Key interventions included antiepileptic therapy for seizure control, osmotic agents to manage intracranial hypertension, and critical care support when necessary.

In transplant recipients, vigilant assessment enables the early detection of complications, such as secondary infections and organ failure, allowing for timely therapeutic adjustments. Additionally, a thorough pretransplant screening of both donors and recipients is essential to reduce the risk of dengue transmission in this high-risk population. Since advanced organ failure in these patients often coincides with significant multisystem involvement, integrating comprehensive clinical evaluation with appropriate diagnostic testing becomes even more critical to ensure transplant safety and optimize posttransplant outcomes. 8 9

In conclusion, dengue encephalitis is a severe and uncommon manifestation of the infection that must be recognized early and diagnosed using appropriate laboratory and imaging tools to provide adequate support to our patients. Although no specific antiviral therapy is currently available, timely supportive care and vigilant monitoring can significantly influence clinical outcomes.
